# Efficacy of synergistic nursing and flexible play-based approach in children with Bronchial Asthma

**DOI:** 10.12669/pjms.41.12.12626

**Published:** 2025-12

**Authors:** Qianli Guo, Baochi Li, Yuxiao Hu, Linlin Liu, Wenshan Lv

**Affiliations:** 1 Qianli Guo, Department of Respiratory, Children’s Hospital of Hebei Province, Shijiazhuang, Hebei Province 050000, P.R. China; 2 Baochi Li, Department of Respiratory, Children’s Hospital of Hebei Province, Shijiazhuang, Hebei Province 050000, P.R. China; 3 Yuxiao Hu, Department of Respiratory, Children’s Hospital of Hebei Province, Shijiazhuang, Hebei Province 050000, P.R. China; 4 Linlin Liu, Department of Respiratory, Children’s Hospital of Hebei Province, Shijiazhuang, Hebei Province 050000, P.R. China; 5 Wenshan Lv, Department of Respiratory, Children’s Hospital of Hebei Province, Shijiazhuang, Hebei Province 050000, P.R. China

**Keywords:** Bronchial asthma, Collaborative care, Games, Negative emotions, Quality of life

## Abstract

**Objective:**

To explore the efficacy of synergistic nursing and flexible play-based mode in children with bronchial asthma.

**Methodology:**

The clinical data of 120 children with bronchial asthma, treated in Children’s Hospital of Hebei Province from February 2023 to April 2025, were retrospectively selected. Based on the nursing approach, patients were divided into the control (routine nursing) and the study group (synergistic nursing and flexible play-based mode in addition to the routine nursing). The recovery RATE, negative emotions before and after intervention (assessed using Screen for Child Anxiety Related Disorders [SCARED] and Depression Self-Rating Scale for Children [DSRSC], quality of life (assessed by Asthma Quality of Life questionnaire [AQLQ]) and nursing satisfaction of the family members were compared.

**Results:**

The duration of wheezing, the disappearance of cough episodes and the hospitalization time were significantly shorter in the study group than in the control group (P<0.05). After intervention, the SCARED and DSRSC scores in the two groups were considerably lower than before intervention (P<0.05). The scores of emotional function, restricted activity, environmental irritation and symptoms in the two groups after intervention were higher than preintervention and markedly higher in the study group compared to the control group (P<0.05). The nursing satisfaction of the family members of the study group (95.24%) was higher than that of the control group (82.46%) and the difference was statistically significant (P<0.05).

**Conclusion:**

Synergistic nursing and flexible play-based mode in children with bronchial asthma can shorten the time of symptom relief, alleviate negative emotions, improve the quality of life, promote the early recovery and discharge of children and is associated with higher family satisfaction levels.

## INTRODUCTION

Bronchial asthma (BA) is a common respiratory disease that affects up to 2-10% of children.^1^ In pediatric patients, this prolonged and recurring disease affects lung function and is associated with psychological stress response (such as anxiety and fear) and impaired quality of life.^2^-^4^ Decreased treatment compliance remains a serious issue in pediatric BA patients, with some studies reporting mean adherence rates below 50%.^5^ Non-adherence to prescribed BA treatment regimens can lead to poor symptom control and higher rates of exacerbations.^6^

Nurses play a crucial role in the control and treatment of BA and in empowering patients with asthma to self-manage the disease. Traditional nursing approaches help standardize asthma management. However, they often overlook children’s unique physical and psychological needs, particularly in reducing negative emotions and encouraging active cooperation.^7^,^8^

As the “second language” of children, play functions as a psychological soothing and cognitive guidance aid that can distract the child from the treatment, reduce the anxiety of operation and help to improve the disease knowledge through role-playing.^9^,^10^ The synergistic care model emphasizes tripartite collaboration between nursing personnel, children and their families and improves quality of care through standardized paths and personalized guidance.^11^,^12^ The dynamic mode of “evaluation-game intervention-cooperative intervention-effect feedback” is built by deeply integrating the game elements with the collaborative nursing process, which can break through the limitations of traditional nursing. Such an approach improves education and treatment cooperation by utilizing the game’s situational characteristics, enhances children’s subjective initiative and strengthens the caregiver’s disease management ability through the family participation game, forming a continuous intervention synergy.^13^,^14^

At present, the clinical research on play-oriented and family-centered nursing approaches in asthma care is still developing. However, recent high-quality international studies have provided growing evidence for their effectiveness. A 2025 systematic review and meta-analysis by Akca Sumengen A et al.^15^ confirmed that game-based asthma interventions significantly improve pediatric asthma control, knowledge, and reduce hospitalizations and emergency visits. Similarly, Karakul A et al.^16^ demonstrated in a 2024 randomized trial that mobile game training enhanced asthma self-management and quality of life in children. Samady W et al.^17^ also validated the impact of interactive inpatient asthma education through a controlled trial design. Beyond play-based interventions, recent studies have emphasized the role of family engagement and digital support tools. A multicomponent school-based intervention trial by Reznik M et al.^18^ showed improvements in asthma control and adherence, while Mosnaim GS et al.^19^ reviewed the application of digital inhalers and remote monitoring, and proposed a framework for digital health integration in asthma care.^20^

This study aimed to build upon these findings by exploring a synergistic model that combines standardized collaborative care with flexible, age-specific play-based interventions for children with bronchial asthma.

## METHODOLOGY

The clinical data of 120 children with bronchial asthma, treated in our hospital from February 2023 to April 2025, were retrospectively selected. Children who received the routine nursing comprised the control group (n=57), while children in the study group (n=63) received synergistic nursing and a flexible play-based mode of care in addition to the routine nursing.

### Ethical approval:

This study was approved by our hospital ethics committee with the number 202222-20, Date: July 4^th^ 2022. Written informed consent was obtained from the legal guardians of all participants, and age-appropriate assent was secured from children capable of understanding the procedures and interventions involved in the study.

### Inclusion criteria:

- Meet the diagnostic criteria^21^ for bronchial asthma.
- 3~12 years old.
- Have basic language understanding and communication skills (such as completing simple instructions or game interaction).
- Complete clinical data.

### Exclusion criteria:

- Severe organ dysfunction such as heart, liver and kidney, or life-threatening acute severe condition such as asthma status and respiratory failure.
- Psychological diseases that affect game participation and nursing cooperation, such as autism, intellectual disability, severe anxiety and depression.
- A clear history of allergies to game materials (e.g., specific toys, pigments) or intervention drugs (e.g., bronchodilators) used in the study
- Chronic diseases that may mask asthma symptoms, such as congenital respiratory tract deformity and cystic fibrosis.

### Routine nursing care (control group):

- *Drug treatment:* Patients were instructed to strictly follow the doctor’s advice to carry out atomized inhalation (such as budesonide + salbutamol), oral leukotriene receptor antagonist and observe the adverse reactions (such as hand shaking and pharyngeal discomfort).
- *Disease monitoring:* Cough frequency, wheezing degree and oxygen saturation (SpO_2_) were recorded every day and pulmonary function indexes (FEV 1 and PEF) were monitored every four hours during an acute attack.
- *Environmental management:* The ward is kept at 18~22°C, humidity 50 %~60%, avoiding flowers and soft toys and is regularly ventilated and disinfected.
- *Psychological care:* The emotional changes of the children are observed and the tension is relieved through soothing language (such as “baby will soon get better with treatment”). The psychological counselor is called to intervene if necessary.
- *Dietary guidance:* Patients are instructed to avoid allergy foods (e.g., seafood, nuts), recommended a high-protein, high-vitamin diet (e.g., eggs, fresh vegetables and fruits) and encouraged to eat a small number of meals in the acute phase to avoid overfullness-induced cough.

All routine nursing measures were delivered by licensed pediatric nurses who had completed standardized training in asthma care. Both the control and intervention groups received the same frequency and intensity of nursing interventions during hospitalization. The only difference between the two groups was the addition of synergistic nursing and play-based modules in the intervention group.

### Synergistic nursing and flexible play-based approach (study group):

Synergistic nursing and play are implemented in addition to routine nursing. (Synergistic nursing system (medical care-children-family tripartite cooperation):

### Multidisciplinary team:

The team includes responsible nurses (leading game intervention), respiratory physicians, child psychologists, rehabilitation therapists and nutritionists. Once a week, the team carries out combined rounds. The team adjusts the game plan and nursing objectives according to the children’s condition (such as acute attack period vs maintenance period) and age. The psychologists assess the degree of negative emotions and formulate a personalized psychological guidance plan.

### Family participation training:

Teaching of nursing skill game, designing the game of “asthma nursing small class”. The guardian practices through role-playing (such as simulating the atomization of dolls) and the nurse corrects the errors on site and issues the sticker of “nursing small expert”. A family WeChat group is set, with one short video released daily (such as “three steps to clean the nebulizer correctly”). The guardian is asked to upload the operation video and punch the card and the nurse gives feedback and guidance within 24 hours. “Family Asthma Diary” is implemented to record the work. The children are instructed to record the condition with pictures (such as stars indicating no cough today). The guardian records the condition with words and the nurse reviews and rewards (such as 10 points for small toys) the participants every week.

### Flexible play-based mode of care (age-specific design):

Young group (3~6 years old): Conduct situational role-playing game to alleviate treatment fear, establish medication compliance and enhance the interest of respiratory training, specific protocol: A. Nebulization: Decorate the nebulization protective screen with cartoon sticker (such as the animated character that the child likes), name it “magic respirator,” and tell the child that “inhaling magic smoke can defeat the small monster in the lung”; Play the children’s song “Deep Breathing Song” (lyrics: “Inhaling like smelling small flowers, exhaling like blowing candles”), guide the child to complete the atomization with the breathing rhythm and give a sticker reward after each completion. B. Small theatre for asthma prevention: The scene is simulated with doll props: “The baby bear is allergic to peanuts, the asthma attacks → the doctor treats with nebulizers → the baby bear learns to stay away from allergens after recovery,” so that the child can participate in the plot interpretation and strengthen the cognition of “avoiding contact with allergens.” C. Breathing training game: Conduct a bubble blowing competition and practice abdominal breathing through bubble blowing. The nurse demonstrates “slowly inhale the belly and gently exhale the bubble to make it fly far.” The child will be praised orally whenever the bubble is blown out (“Wow, your bubble is flying high!”).

### School age group (7~12 years old):

Carry out cognitive games to improve the mastery of disease knowledge, cultivate self-management ability and enhance the awareness of family cooperation. Specific scheme: A. Rich asthmatic knowledge: Design a checkerboard game, the grid content includes the causes of asthma (such as “meet pollen → back 3 steps”), correct treatment methods (such as “carry emergency medicine with you → forward 5 steps”), children and guardians team to participate, get points for correct questions and finally the group with the highest points gets the certificate of “asthma management person.” B.” My asthma” action plan card preparation: Provide a colorful pen and a card to guide the child to draw “3 steps during an asthma attack” (such as sitting down for rest, inhaling medicine, calling his/her father and mother). The card can be carried with him/her. The nurse regularly checks and supplements the contents (such as adding a newly recognized allergen picture). C. Family Sports Challenge: Design “asthma-friendly” sports games (such as jumping rope and kicking shuttlecock), set the sports target every week (such as 20 minutes of exercise every day), the children and their guardians complete the exercise, record the exercise duration through the mobile phone APP, unlock the “Sport Medal” to complete the target and convert the accumulated medal into the library loan card, stationery and other prizes.

### Dynamic Assessment and Protocol Adjustment:

**Effect feedback mechanism:**

After daily intervention, the children’s game participation (such as “actively completing the atomization game/urging parents”) is recorded and emotional reaction (such as “smiling/crying”) is noted. The C-ACT score and anxiety scale are retested every week. A family feedback meeting is conducted to share the “favorite game” and “the place where you find it difficult,” the guardian puts forward nursing suggestions and the group adjusts the content of the next stage of the game according to the feedback (such as adding the theme role that the child is interested in).

### Emergency treatment fusion:

Embed an emergency scenario simulation in the game. For example, the young group teaches children to call “mother, I need medicine” through the game “Little Bear’s Asthma Attack.” The school-age group strengthens the process of “not panicking during acute attack, taking medicine first and then seeking medical treatment” through knowledge questions and answers. In both groups, the intervention lasted for four weeks.

### Observation indexes:

- Recovery was evaluated for both groups, including wheezing, cough resolution time and hospital stay.
- Negative emotions were counted in two groups before and after the intervention. Children’s Anxiety Mood Disorder Screening Scale (SCARED) and children’s Depressive Disorder Self-Rating Scale (DSRSC) were adopted, respectively. SCARED included forty-one items and a three-grade score was adopted. The score range was 0~ 82. The higher score indicated more serious anxiety. The DSRSC consisted of eighteen items assessed by a three-level scoring method with a score range of 0~ 36. A higher score indicated more severe depression.
- The quality of life before and after intervention was counted in two groups using the Asthma Quality of Life Questionnaire (AQLQ), including emotional function, limited activity, environmental irritation and symptoms. Thirty-two items were included. The score of each item ranged from 1~ 7. A higher score indicated better quality of life.
- The nursing satisfaction of the family members in the two groups was counted and assessed by the Newcastle Nursing Satisfaction Scale (NSNS), with a total possible score of 95 points. Scores below 67 indicate dissatisfaction, scores ranging from 67 to 85 reflect moderate satisfaction and scores above 85 signify high satisfaction. Both moderate and high satisfaction scores were included in the overall satisfaction score.

### Observation indexes:

Throughout the intervention period, safety and adherence were monitored by nursing staff through daily observations and WeChat-based family interaction logs. No serious adverse events (e.g., physical injury, hospitalization, or severe emotional distress) occurred. Mild resistance behaviors (e.g., crying, refusal to cooperate) were occasionally observed, particularly during initial exposure to new game tasks, but resolved spontaneously or after verbal reassurance. Parental adherence was evaluated based on task completion (e.g., video uploads, asthma diary entries). Occasional non-compliance (e.g., missed check-ins) occurred due to scheduling conflicts or forgetfulness. Nurses provided timely follow-up and individual guidance through digital communication.

### Statistical analysis:

Data were analyzed by SPSS 25.0. Measurements data was expressed as (±s) and the *t* test was used; counting data was presented as frequency and composition ratio (%) and the *χ^2^* test was used. Prior to applying t-tests, the Shapiro–Wilk test was used to assess the normality of continuous variables, and Levene’s test was conducted to evaluate the homogeneity of variances. All variables used in t-tests satisfied the assumptions. For variables that did not meet these assumptions (none in the final analysis), non-parametric alternatives such as the Mann–Whitney U test were considered. P<0.05 indicates a statistically significant difference.

## RESULTS

There was no significant difference between the two groups in gender, age, course of disease, degree of disease, education background of main caregivers, place of residence, physical exercise and other general characteristics (P>0.05). Table-I

**Table-I:** General characteristics of the two groups.

Item	Study Group (n=63)	Control Group (n=57)	t/χ^2^ Value	P Value
***Gender[n(%)]***				
Male	35(55.56)	34(59.65)	0.205	0.651
Female	28(44.44)	23(40.35)		
Age(±s, year)	7.79±2.38	8.02±2.54	0.512	0.610
Course of diseases(*Χ̅±S*, year)	3.22±1.37	3.05±1.56	0.636	0.526
***Severity [n(%)]***				
Mild	25(39.68)	24(42.11)	0.630	0.730
Moderate	27(42.86)	26(45.61)		
Severe	11(17.46)	7(12.28)		
***Primary caregiver’s educational level [n(%)]***			
Primary school and Junior high school	13(20.63)	14(24.56)	1.391	0.499
Senior and Vocational high school	36(57.14)	35(61.40)		
Associate Degree or higher	14(22.22)	8(14.04)		
***Location of Residence[n(%)]***				
Urban	41(65.08)	39(68.42)	0.150	0.698
Rural	22(34.92)	18(31.58)		
***Physical Exercise [n(%)]***				
Hardly	19(30.16)	16(28.07)	0.423	0.809
1~3times every week	24(38.10)	25(43.86)		
Every week>3times	20(31.75)	16(28.07)		

Time to wheezing resolution and disappearance, time to cough disappearance and the length of hospitalization in the study group were considerably shorter than in the control group (*P*<0.05) is shown in Table-II.

**Table-II:** Comparison of rehabilitation between two groups (*Χ̅±S*, d).

Group	Number of cases	Time to Wheezing Resolution	Time to Wheezing Disappearance	Time to Cough Resolution	Hospital Stay Duration
Study Group	63	4.17±1.15	3.28±1.27	3.08±1.36	5.59±1.62
Control Group	57	6.32±1.59	5.11±1.64	4.82±1.22	6.99±1.47
*t* Value		8.545	6.869	7.348	4.939
*P* Value		0.000	0.000	0.000	0.000

There was no significant difference in SCARED and DSRSC scores between the two groups before intervention (P>0.05). After the intervention, the SCARED and DSRSC scores of the two groups were lower than those before the intervention and significantly lower in the study group compared to the control group (P<0.05; **Table-III**). Similarly in the scores of emotion function, restricted activity, environmental stimulation and symptoms between the two groups before intervention (P>0.05). The scores of emotion function, restricted activity, environmental stimulation and symptoms in the two groups after intervention were markedly higher than before intervention and significantly higher in the study group compared to the control group (P<0.05; Table-IV).

**Table-III:** Comparison of negative emotions (*Χ̅±S*, min).

Period	Group	Number of cases	SCARED	DSRSC
Before intervention	Study Group	63	29.64±3.17	19.76±3.34
	Control Group	57	30.53±2.94	20.21±3.02
	*t* Value		1.589	0.771
	*P* Value		0.115	0.442
After intervention	Study Group	63	16.51±2.81	9.91±2.17
	Control Group	57	19.74±3.07	11.86±2.51
	*t* Value		6.018	4.563
	*P* Value		0.000	0.000

**Table-IV:** Comparison of QOL between two groups (*Χ̅±S*, min).

Period	Group	Number of cases	Emotional function	Restricted activity	Environmental irritation	Symptoms
Before intervention	Study Group	63	16.69±3.11	43.24±5.17	12.84±4.02	47.74±6.80
	Control Group	57	17.13±3.37	42.61±5.64	13.15±3.71	46.81±5.84
	t Value		0.744	0.638	0.438	0.800
	P Value		0.459	0.524	0.663	0.426
After intervention	Study Group	63	24.91±3.42	59.91±4.55	19.15±3.80	64.09±7.02
	Control Group	57	21.89±3.60	55.72±3.83	17.07±4.05	59.66±6.64
	t Value		4.711	5.427	2.902	3.542
	P Value		0.000	0.000	0.004	0.001

The nursing satisfaction of the family members of the study group (95.24%) was higher than that of the control group (82.46%) and the difference was statistically significant (*P*<0.05). Table-V

**Table-V:** Comparison of nursing satisfaction between two groups[n(%)].

Group	Number of cases	Very Satisfied	Moderately Satisfied	Dissatisfied	Overall Satisfaction
Study Group	63	38(60.32)	22(34.92)	3(4.76)	60(95.24)
Control Group	57	29(50.88)	18(31.58)	10(17.54)	47(82.46)
*χ^2^Value*					5.061
*P Value*					0.024

## DISCUSSION

This study aimed to explore the efficacy of synergistic nursing and flexible play-based approach on symptom relief, negative emotions, quality of life in children with BA and to evaluate family satisfaction with the nursing care. The results showed that the combined approach was more effective in improving symptoms, such as wheezing and cough and shortening hospitalization time, indicating that this nursing mode can accelerate the recovery process of children with bronchial asthma.

The traditional nursing approach is often not enough to improve cooperation and family participation in children with bronchial asthma, who often suffer from psychological resistance due to the long treatment cycle and complicated operation.^22^ The results of this study are consistent with the conclusion of Poot CC et al.^23^ that “play-based nursing can improve the treatment compliance and ensure the recovery effect of the disease.” Specifically, the game intervention reduces the treatment fear through scene simulation. In the “magic respirator” game, the atomized protective screen is imagined as the weapon to defeat the monster. School-age children strengthen their medication memory through the game of “asthma knowledge breakthrough,” which can improve the degree of cooperation and ensure the disease recovery effect.

At the same time, the multi-disciplinary team of collaborative care dynamically adjusts the program every week (such as focusing on anxiety relief in the acute phase and intensifying breathing training in maintenance phase) to avoid the lag of traditional care. This is consistent with the viewpoint of Andrea Reupert and others^24^ that “collaborative care can optimize the whole-course management of disease.” Each week, the multidisciplinary care team—including clinicians, family members, and rehabilitation specialists—conducts case discussions. Physicians adjust medication dosage based on game-based feedback, such as children’s resistance to nebulization. Nurses refine the workflow by tracking measurable indicators like the number of correct uses of the mist inhaler. At the same time, the family members learn the observation points of the condition in the game (such as mastering the recognition of abnormal signs through the game of “respiratory rate counter”), thus ensuring the overall rehabilitation quality.

After intervention, the scores of SCARED and DSRSC in the study group were significantly lower than those in the control group (P<0.05), suggesting that the synergistic nursing and flexible play mode had significant advantages in relieving anxiety and depression in children with BA. The study by Nora Suleiman-Martos N et al.^25^ indicated that play therapy can reduce the anxiety of medical operations in children, consistent with this study’s results. Moreover, in terms of clinical significance, previous studies have reported a Minimal Clinically Important Difference (MCID) of approximately 8–9 points for the SCARED scale and 4–5 points for the DSRSC scale.^26^-^28^ In our study, the intervention group exhibited a mean SCARED score reduction of 13.13 points and a DSRSC reduction of 9.85 points, both of which exceeded the reported MCID thresholds. These findings indicate that the observed improvements were not only statistically significant but also clinically meaningful. Through the doll theatre of “Little Bear Asthma Attack,” the psychological desensitization of the disease was completed in the role play of the young children. The children of school age express their emotions in the drawing of “Family Asthma Diary,” and their parents interact with each other online, forming a dual pathway of “game-family support.” Compared with the routine psychological intervention of traditional nursing, the synergistic play-oriented approach more effectively breaks the psychological defense mechanism of the children through active participation in the game and the emotional involvement of the family.

This approach’s family support system is the key link to emotional improvement. For instance, the psychologist may instruct the parents to use the “empathetic dialogue” in the “family emotional theatre” game to improve the efficiency of parent-child communication. The dietitian may develop a diet plan with the children through the “food magic game,” converting the “refusing to take medicine” into “accomplishing the health task”. Together, these measures may significantly increase family participation. Overall, the synergistic mode of “medical care guidance-family execution-game enabling” constructs a more three-dimensional support network than traditional psychological care.^29^,^30^

This study demonstrated that after intervention, the scores of each dimension of AQLQ and the satisfaction of family members in the study group were significantly higher than those in the control group (P<0.05), indicating that the synergistic nursing and the flexible play mode can effectively improve the life quality of children with BA and deepen the degree of acceptance of the nursing mode by the family members. It is plausible that the synergistic nursing and flexible play mode can increase the interest in nursing work, improve children’s compliance and ensure the early recovery of clinical symptoms, thus improving the treatment effect. At the same time, the remission rate of symptoms in children with bronchial asthma intuitively reflects the effectiveness of the nursing plan. Therefore, it enhances the trust of the family members and increases the family’s satisfaction with the nursing care.

Importantly, no serious adverse events were reported during the intervention period, supporting the safety of the play-based synergistic care model. Although occasional mild behavioral resistance (e.g., crying or refusal to join activities) was noted, these were transient and manageable through nurse engagement. Minor adherence lapses among parents (e.g., incomplete task submissions) were addressed via real-time WeChat follow-up. Future studies should incorporate standardized adherence monitoring systems, such as mobile app check-ins or structured adherence scales, to improve replicability and long-term tracking.

In contrast to existing studies^16^,^17^ that often utilize play interventions in isolated clinical settings (e.g., pre-nebulization distraction), our study contributes a novel, integrative approach by embedding flexible, age-stratified game content within a multidisciplinary synergistic nursing model. This model extends across hospitalization and post-discharge phases, forming a continuous and closed-loop intervention pathway. Moreover, by incorporating structured family engagement mechanisms—such as home-based task tracking, real-time WeChat feedback, and personalized game adaptation—this study addresses key barriers in pediatric asthma care, particularly poor treatment adherence and limited caregiver involvement. The dynamic design combining clinical assessment, caregiver execution, and play-based behavioral reinforcement represents a practical innovation with potential for broader application in chronic pediatric disease management. Compared with previous studies^18^,^19^, our findings extend the current evidence base on play-based asthma care by integrating these interventions within a structured, multidisciplinary collaborative model. While prior RCTs and reviews have validated play therapy or digital tools as standalone strategies, few have explored their integration within a standardized nursing framework that also emphasizes family engagement. Our study contributes to this growing body of research by demonstrating the feasibility and clinical benefit of combining collaborative care with age-appropriate play in pediatric asthma management.

### Limitations:

First, it was a single-center retrospective observational study with a moderate sample size (n=120), which may introduce selection bias and limit the generalizability of the findings. The lack of randomization also restricts the ability to draw causal inferences regarding the observed outcomes. Second, the intervention period was limited to four weeks, focusing on short-term symptom relief and psychological improvements during hospitalization. As a result, the durability and long-term effectiveness of the intervention remain uncertain.

## CONCLUSION

The synergistic nursing and play-based care model significantly improves clinical symptoms, emotional well-being, and quality of life in children with asthma. It also increases family satisfaction and offers an innovative, practical approach for pediatric chronic disease management.

### Future recommendations:

Future studies should employ multicenter, prospective randomized controlled designs with extended follow-up to assess sustained outcomes such as recurrence rates, exacerbation frequency, and long-term adherence. Third, although the intervention appeared to enhance treatment adherence based on nurse observations and caregiver reports, no objective adherence measures were implemented. The absence of tools such as medication usage logs, post-discharge pulmonary function tests (e.g., FEV_1_ or PEF), or validated symptom control instruments (e.g., Childhood Asthma Control Test [C-ACT]) limits the ability to quantify adherence improvements. Future research should incorporate these metrics to enhance the robustness of adherence assessment. Fourth, potential response bias may have influenced the family satisfaction results. While we used the validated Newcastle Satisfaction with Nursing Scale (NSNS), the non-anonymous administration and in-hospital setting might have discouraged participants from expressing negative opinions. Future assessments should consider anonymous electronic questionnaires or third-party-administered surveys to improve data objectivity and reliability. Fifth, variability in family engagement remains a relevant concern. Although baseline comparisons showed no significant differences in caregiver education or urban–rural residence, unmeasured factors such as parenting attitudes, socioeconomic capacity, and health literacy may have influenced the quality of participation and, consequently, intervention efficacy. Future studies may benefit from stratified or subgroup analyses to better understand how these contextual factors affect outcomes. Finally, the current game-based intervention lacked standardized content and delivery procedures, which may affect reproducibility. Future research should aim to develop unified, scalable game protocols. In addition, integrating digital health technologies—such as AI-powered asthma apps, telehealth platforms, and VR-based therapeutic games—may enhance personalization, engagement, and continuity of care across hospital-to-home transitions.

### Funding:

Hebei Province 2023 Medical Science Research Project Plan (20231139).

### Authors’ contributions:

**QG:** Study design. literature search and manuscript writing.

**YH, LL, WL and BL:** Data collection, data analysis, interpretation and critical review.

**QG:** Was involved in the manuscript revision and validation and is responsible for the integrity of the study. All authors have read and approved the final manuscript.
